# Towards new definitions of avoidable hospital admissions

**DOI:** 10.3399/bjgp22X720725

**Published:** 2022-10-30

**Authors:** Matthew Booker, Sarah Purdy

**Affiliations:** Centre for Academic Primary Care, Population Health Sciences, University of Bristol, Bristol.; Centre for Academic Primary Care, Population Health Sciences, University of Bristol, Bristol.

An unscheduled hospital admission often represents a major upheaval, with potential for physical, psychological, social, and economic consequences. Hospital admissions are also associated with an increased risk of adverse consequences to both physical and emotional wellbeing. Indeed, the broader negative impacts of the ‘allostatic stress’ of an admission may even outstrip those of physical illness in depleting reserves — a risk that persists beyond discharge.^1^ In patients with frailty, even a short so-termed ‘ambulatory’ admission is associated with increased mortality and subsequent use of health resources.^2^

While a hospital admission is rarely relished, there is evidence across international health systems that the COVID-19 pandemic has heightened peoples’ desire to stay out of hospital, with an increasing trend of patients delaying or avoiding seeking urgent care altogether for fear of being admitted.^3^ While specific worries about hospital-acquired infection are undoubtedly a component, it appears that people may be more broadly re-evaluating the pros and cons of unscheduled hospital care as part of their re-framed priorities in the post-pandemic era. These new sets of priorities, combined with a renewed urgency (driven by unprecedented demand) to explore alternative models of unscheduled care that don’t require inpatient stays, mean we may need to look again at how we conceptualise the avoidable admission.

A major challenge of research in this area is the lack of a single, consistently applied definition of an avoidable admission.^4^ Academics and clinicians alike have long sought a concise and utilitarian way to define exactly who these patients might be, and in which circumstances alternative care is practical and appropriate.

One approach is to define this cohort according to a disease or illness for which there exists a viable care pathway that does not require an inpatient stay. Ambulatory care sensitive conditions (ACSCs) are one such classification, defining conditions where effective person-centred community care may prevent the need for hospital admission.^5^ Along with others, we have previously utilised a nuanced version of this definition — primary care sensitive conditions (PCSCs) — in which the list of conditions is extended to include ‘situations’ that may not themselves be diagnoses or illnesses, but which may be amenable to timely and holistic primary care input to avoid an admission (for example, social care crises).^6^ Similarly, the term urgent care sensitive conditions (UCSCs) has been used in the literature to describe when same-day urgent care may prevent further resource use,^7^ although the definition of urgent care is not itself universally agreed.

Basing the study of avoidable admissions on ASCSs alone, however, results in an incomplete understanding of the phenomenon. Recent analysis identified a complex relationship between ACSCs, admissions, and ‘preventable’ emergency care.^8^ The potential ability of primary care to decrease the number of admissions due to ACSCs (and PCSCs/UCSCs) is confounded by the sheer variation in the way services are delivered^9^ — heterogeneity, which has been further compounded by the COVID-19 pandemic. Even the relationship between availability of GPs and emergency admissions is not straightforward,^10^ suggesting that it is not just a primary care capacity issue.

## AVOIDABLE, UNNECESSARY, AND INAPPROPRIATE ADMISSIONS — ONE AND THE SAME?

These terms are often used interchangeably in both academic and health policy literature, although each is describing something conceptually discrete. Indeed, it is possible for a non-elective hospital admission to be any one or combination of the above, or none. The context in which the need arises is intrinsically linked to how these definitions can be applied.

The majority of policy solutions to rising numbers of unscheduled hospital admissions rest in one of three related assumptions: 1) a significant proportion of urgent health care is being delivered in a suboptimally efficient manner, setting, or format; 2) better patient-centred outcomes can be achieved by delivering care outside hospital; and 3) a sizeable proportion of unscheduled admissions achieve little or no net benefit.

One recent approach to emergency department visits has been to conceptually separate attendances that are ‘clinically preventable’, ‘clinically divertible’, and ‘clinically unnecessary’,^11^ recognising that these are distinct patient groups whose outcomes are likely to be influenced by different intervention designs and the operation of different mechanisms. While it is important to recognise that there are multiple routes into an acute hospital bed not all of which pass through the emergency department, the same conceptual classification can extend more broadly to admissions as a whole.

Each of these require us to make some potentially challenging judgements. For example, divertible cases require a viable alternative to be operational at the point of need, with adequate capacity to respond, else they cease to be divertible. Policies of re-direction are not without their critics, as there can be major consequences if this diversion requires more resource from an already overwhelmed primary care service. There also exists a rich discourse on the problems with terming healthcare use as ‘inappropriate’. The literature is light on understanding urgent and emergency care resource use decisions from the patient perspective: ‘inappropriate’ is almost always a retrospective label applied from a service-centric perspective.

## THE NON-BENEFICIAL ADMISSION

More recently the concept of a ‘non-beneficial’ admission has entered the discourse more widely. Seen by some as a more patient-centric term, it is a nuanced extension of the broader concept of ‘futile’ or non-beneficial treatment. With respect to hospital admissions, it has been defined as when a patient is already known to have an incurable condition beyond the ability of the hospital to give net benefit, which can and should be treated outside hospital.^12^

While this definition is helpful in ensuring the purpose of an admission is kept in the forefront of the mind, it too is context and situation specific. Benefit from admissions may be broad, may be psychosocial (at least in part), and may be the result of a balancing act of limited or suboptimal alternatives. Benefit may also be defined from a range of perspectives.

Exploring all of these definitions of unscheduled hospital admissions highlights the paradox. Being admitted carries a risk of adverse consequences. For some groups in society these risks are serious, enduring, and undesirable. Yet, hospital is sometimes the only practical setting where potentially beneficial interventions can take place, particularly in a context that cannot support a viable alternative at the point it is needed. Indeed, failing to explore and, where appropriate, challenge a disproportionate aversion to hospital admission for a highly treatable or reversible condition does not constitute ‘good medicine’.

## TOWARDS FOCUSED ADMISSIONS

Hospital care is a necessary, resource-appropriate, and valued part of many acute medical and surgical situations. Yet, in practice, the consequences of the ‘admission’ and the benefits of the ‘intervention’ are often hard to disentangle.

This very issue colours the way hospital admission avoidance interventions are evaluated. When the aim is preventing an inpatient stay at all costs, it is possible to overlook the value of many interventions that mitigate some of the negative consequences of an unscheduled admission, even if it is not completely avoided. Being optimised for a shorter, strategic hospital stay for a defined procedure or investigation is not necessarily a ‘failure’ of a hospital-at-home service, yet some would consider that it hasn’t achieved its objective. Conversely, the suggestion that keeping an end-of-life patient who would never have been a candidate for a hospital stay at home is a ‘success’ of admission avoidance is probably disingenuous.

In the light of the refreshed priorities, it might be time to consider the concept of the focused admission — one that involves the shortest possible stay, with a clearly defined objective that provides ‘net benefit’ when viewed from the patient perspective. As the specific location of the care becomes less of a focus than the objective, the boundaries between community- and hospital-based care may become less fiercely defended by both sides.

## References

[1] Krumholz HM (2013). Post-hospital syndrome — an acquired, transient condition of generalized risk. N Engl J Med.

[2] Keeble E, Roberts HC, Williams CD (2019). Outcomes of hospital admissions among frail older people: a 2-year cohort study. Br J Gen Pract.

[3] Howley F, Lavan A, Connolly E (2021). Trends in emergency department use by older people during the COVID-19 pandemic. Eur Geriatr Med.

[4] Purdy S, Hunley A (2013). Predicting and preventing avoidable admissions: a review. J R Coll Physicians Edinb.

[5] Purdy S, Griffin T, Salisbury C, Sharp D (2009). Ambulatory care sensitive conditions: terminology and disease coding need to be more specific to aid policy makers and clinicians. Public Health.

[6] Booker MJ, Shaw ARG, Purdy S (2015). Why do patients with ‘primary care sensitive’ problems access ambulance services? A systematic mapping review of the literature. BMJ Open.

[7] Nuffield Trust (2022). Potentially preventable emergency admissions. https://www.nuffieldtrust.org.uk/resource/potentially-preventable-emergency-hospital-admissions.

[8] Frick J, Möckel M, Muller R (2017). Suitability of current definitions of ambulatory care sensitive conditions for research in emergency department patients: a secondary health data analysis. BMJ Open.

[9] Busby J, Purdy S, Hollingworth W (2017). Opportunities for primary care to reduce hospital admissions: a cross-sectional study of geographical variation. Br J Gen Pract.

[10] Nicodemo C, McCormick B, Wittenberg R, Hobbs FDR (2021). Are more GPs associated with a reduction in emergency hospital admissions? A quantitative study on GP referral in England. Br J Gen Pract.

[11] Parkinson B, Meacock R, Checkland K, Sutton M (2021). How sensitive are avoidable emergency department attendances to primary care quality? Retrospective observational study. BMJ Qual Saf.

[12] Cardona-Morrell M, Kim J, Turner RM (2016). Non-beneficial treatments in hospital at the end of life: a systematic review on the extent of the problem. Int J Qual Health Care.

